# Interprofessional nutrition management – implementation and evaluation of a course for medical and nursing students using research-based learning method

**DOI:** 10.3205/zma001276

**Published:** 2019-11-15

**Authors:** Bärbel Wesselborg, Matthias Hoenen, Renate Adam-Paffrath, Silke Kuske, Lena Schendel, Matthias Grünewald, Stefan Wilm, Thomas Rotthoff

**Affiliations:** 1 Fliedner Fachhochschule Düsseldorf, University of Applied Sciences, Nursing Education, Düsseldorf, Germany; 2 Heinrich Heine University Düsseldorf, Medical Faculty, Deanary of Study, Düsseldorf, Germany; 3 Fliedner Fachhochschule Düsseldorf, University of Applied Sciences, Düsseldorf, Germany; 4 University Hospital Düsseldorf, School for Health Professionals, Düsseldorf, Germany; 5 Heinrich Heine University Düsseldorf, Institute for General Practice, Düsseldorf, Germany; 6 Augsburg University, Faculty of Medicine, Department for Medical Education and Educational Research, Augsburg, Germany

**Keywords:** Interprofessional education, research-based learning, malnutrition, evaluation

## Abstract

**Objective:** The aim of the teaching project “Interprofessional Nutrition Management in Inpatient and Home Care” of the Medical Faculty of the Heinrich Heine University (HHU) and the Fliedner University of Applied Sciences Düsseldorf (FFH) was to test an interprofessional training session on the topic of malnutrition using the method of research-based learning to evaluate feasibility.

**Method: **In the teaching project for medical and nursing students, research-based learning was applied in a case-based cross-sector setting. The teaching project was assessed quantitatively by the participating students through questionnaires and four newly-developed scales. The modeling and reliability of the scales (from 1 to 5) was confirmed by an exploratory factor analysis and Cronbach’s alpha. The scales were evaluated descriptively and through inferential statistics.

**Results: **The medical (n=21) and nursing students (n=25) rated the teaching project positively. Across all professional groups, the social context between the students (M=4.6) and the relevance of the topic (M=4.47) were rated very highly. The use of research-based learning (M=3.9) and the final assessment of the training session (M=3.9) were rated as satisfactory.

**Conclusions: **The method of research-based learning proved to be very suitable for interprofessional education, as it enabled situations which encouraged the health professionals to learn from one another, about one another and with one another. Through the interdisciplinary discussion of malnutrition, cooperation skills and initial competences in nutritional management can be cultivated in future doctors and nursing staff even during training.

## Zusammenfassung

**Zielsetzung: **Ziel des Lehrprojektes „Interprofessionelles Ernährungsmanagement in der stationären und häuslichen Versorgung“ der Medizinischen Fakultät der Heinrich-Heine-Universität (HHU) und der Fliedner Fachhochschule Düsseldorf (FFH) war es eine interprofessionelle Lehrveranstaltung zum Schnittstellenthema Mangelernährung mit der Methode des Forschenden Lernens zu erproben und deren Machbarkeit zu evaluieren.

**Methodik: **In dem Lehrprojekt für Medizin- und Pflegestudierende wurde Forschendes Lernen in einem fallbasierten sektorenübergreifenden Setting angewendet. Das Lehrprojekt wurde quantitativ mittels Fragebogen und vier neuentwickelten Skalen von den teilnehmenden Studierenden bewertet. Die Modellbildung und die Reliabilität der Skalen (Breite 1-5) wurde durch eine explorative Faktorenanalyse und Cronbachs Alpha abgesichert. Die Skalen wurden deskriptiv sowie inferenzstatistisch ausgewertet.

**Ergebnisse: **Die Medizin- (n=21) und Pflegestudierenden (n=25) evaluierten das Lehrprojekt positiv. Berufsgruppenübergreifend wurde das Sozialklima zwischen den Studierenden (M=4,6) und die Relevanz des Themas (M=4,47) sehr hoch eingeschätzt. Die Anwendung des Forschenden Lernens (M=3,9) und die abschließende Einschätzung der Lehrveranstaltung (M=3,9) wurden als zufriedenstellend bewertet.

**Schlussfolgerungen: **Die Methode des Forschenden Lernens erwies sich für interprofessionelle Ausbildung als sehr geeignet, da sie Lernsituationen ermöglichte, in welchen das von-, über- und miteinander Lernen der Gesundheitsberufe gefördert wurde. Durch die professionsübergreifende Auseinandersetzung mit Mangelernährung können bereits in der Ausbildung Kooperationskompetenzen und erste Handlungskompetenzen für Ernährungsmanagement bei den zukünftigen Ärztinnen und Ärzten und Pflegenden angebahnt werden.

## 1. Introduction

For some years now there have been positive developments in the establishment of interprofessional training structures in the health professions in Germany [1]. Among other things, the institutions involved in training health care professionals are taking up the recommendations of the Science Council to offer certain elements of training in an interdisciplinary format [2]. It is proven that interprofessional education (IPE) can improve the quality of patient care as well as the collaboration and job satisfaction of health care professionals [3]. What interprofessional training sessions have in common is a basic understanding that IPE is a process in which different health professionals learn from and about each other to improve their teamworking skills and thus patient- and health care [4]. 

The content of IPE can be configured in various ways. It is possible to make interprofessional co-operation an explicit topic or to practice interprofessionalism using an interdisciplinary topic relevant to the participating health professions [5]. When using interdisciplinary topics in IPE, the aim is to acquire expertise and competencies associated with learning situations [5] which promote learning with, about and from each other among the professions [4]. It is easier to formally embed IPE training sessions into the curriculum using interdisciplinary topics though some challenges may arise due to differing prior knowledge of the health professions involved [5]. In the present project the interdisciplinary topic of malnutrition, which is relevant across health professions, was chosen as an example for the design of IPE. In German hospitals malnutrition remains with a prevalence of more than 25%, with an upward trend which often goes undetected and is not reliably considered in patient treatment [6]. The resulting downstream health and economic consequences are comparatively high and well documented internationally [7].

To date, little experience has been documented regarding the design of IPE using the tertiary education didactic method of research-based learning. Research-based learning aims to guide learners into learning situations in which they actively and independently work on and reflect topics in a cycle of inquiry [8]. Furthermore, there are almost no cross-sector training programs in the health professions that enable the occupational groups to address and deal with problems from multiple perspectives and within context [9].

The aim of the interprofessional teaching project “Interprofessional Nutrition Management in Inpatient and Home Care” was therefore to conduct a cross-sector test of IPE using the method of research-based learning and using the example of the interdisciplinary topic of malnutrition. In the training session evaluation, the assessment of the feasibility and the benefits was to be identified from the students’ perspective in order to obtain reliable results for bespoke future development for both professions. 

## 2. Project description

### 2.1 Positioning of the course

The training session was first held as part of the medical studies at the HHU and in the dual bachelor course nursing and health of the FFH in the summer semester 2017 and in the winter semester 2017/2018 with two semester hours per week in Düsseldorf. In medical studies the course was offered between the 6^th^ and 8^th^ semester as an elective and in nursing studies in the 6^th^ or in the 5^th^ semester as a compulsory course. This seem to be the right point in medical studies because the anatomical, physiological and biochemical basics of nutrition and digestion with selected clinical pictures (3^rd^ and 4^th^ semester) have been completed at this stage. In addition, the students have usually completed in-depth courses in pathology and background to evidence-based medicine (5^th^ and 6^th^ semester). In nursing studies, the topic of malnutrition and nutrition management is completed at both these points in time. 

#### 2.2 Didactic conception of the IPE

Research-based learning can be applied in different didactic formats. For example, it can be used in researching information and facts on a research question and its structured processing and critical discussion; or in the investigation of individual specific problem cases and in conducting case studies using scientific approaches [8], [10]. Research-based learning takes into account a constructivist understanding of learning, is designed systematically and ensures a reflected approach [8]. 

The starting point of research-based learning in this teaching project was exposure to real cases. The cases, hailing from different sectors of the health care system, called for patient care to be optimized through interprofessional action using the example of nutrition management for malnutrition. Through the formation of small interprofessional groups in close cooperation, learning situations were to be created that enabled the two professions [4] to learn with, about and from one another.

#### 2.3 Training session sequence

**Figure 1:**
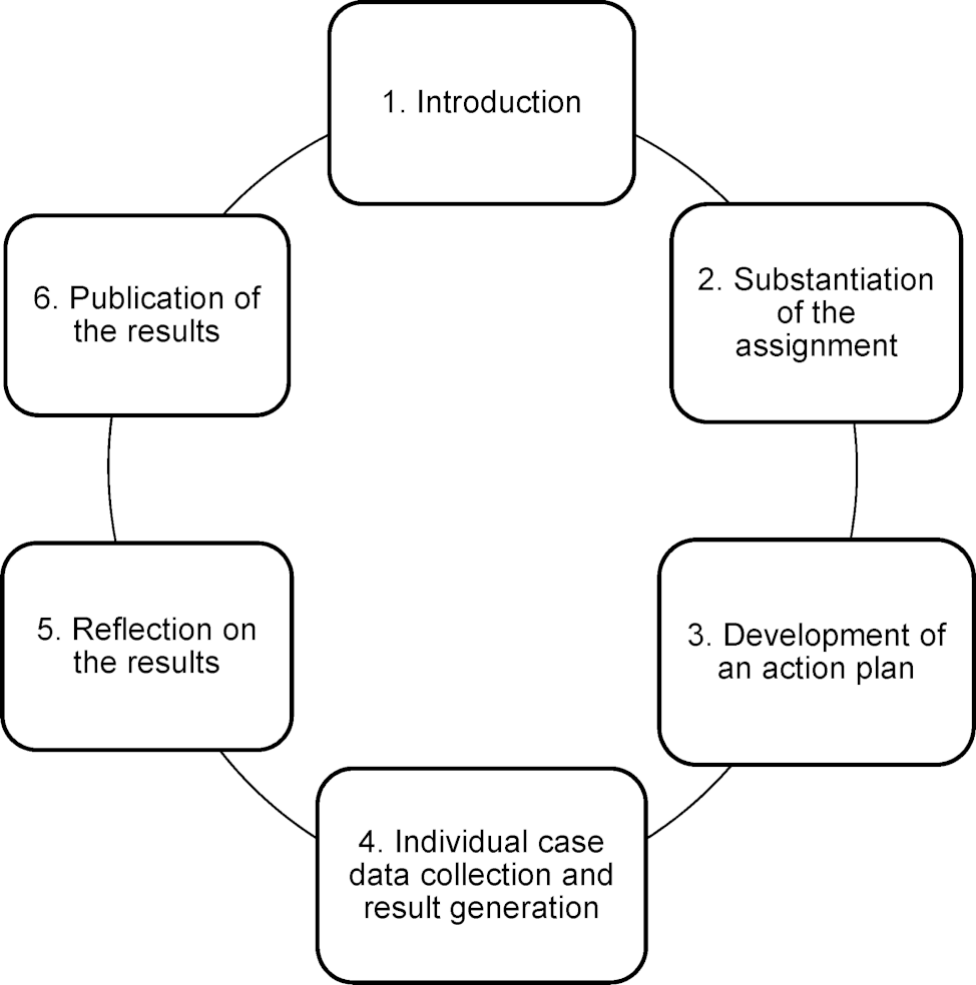
Sequence of the training session based on the phases of research-based learning (own representation in accordance with [10])

The training session followed the phase model of research-based learning and was designed with a block structure to be held on six dates (see figure 1 (Fig. 1)):

1. On the first day of the session, the topic of malnutrition was introduced and the student groups were encouraged to get acquainted with each other. For this purpose, interprofessional teams of three to four students were formed by drawing lots. These were to analyze the nutritional management of patients in the various sectors of the health care system (hospital, out-patient care, primary care) from their various perspectives. These small interprofessional groups exchanged information about their educational and professional background, developed definitions of malnutrition based on their assumptions, and thus initiated a first instance of learning about, from and with one another. Finally, the interprofessional groups worked on key aspects of the causes and therapies for malnutrition, taking into account various clinical pictures.
2. On the second day of the session, the students presented the results of their group work on the various aspects. In addition, various assessment tools were introduced for surveying and rating the nutritional status of a patient and methods for determining nutritional requirements were developed. Subsequently, the knowledge acquired was rehearsed by working through written case studies and information was provided on the planned practical tasks.
3. On the third day of the session, guidelines [11], expert standards [12] and systematic database research on malnutrition were collated and forms of enteral nutrition and aids were discussed in addition. In preparation for the field studies to be carried out, the small interprofessional groups were given the contact details of the areas of practice in the various sectors and the students developed a systematic plan for proceeding.
4. On the fourth day of the session, the students met in their small groups in the various areas of practice. Taking their pre-planning into consideration, they systematically collected the nutritional status of their patients and independently and scientifically developed an interprofessional treatment plan for the case in question. The treatment plan was reported back to the practice partners and with justifications.
5. The fifth day of the session was available to the groups for further elaboration of the results to date, preparation of results posters and reflection and for passing on the results to the teaching staff at the universities.
6. On the closing day, the small interprofessional groups presented their treatment plans to university lecturers, practice partners and institution experts who are responsible for the nutritional status of patients. They used posters to visualize the results of their work and discussed and substantiated the solutions they had developed for their specific cases with scientific data. They also shared their experiences and insights on interprofessional collaboration. They evaluated their professional roles and the part they play in nutrition management.

#### 2.4 Instrument development and sample design

Since literature research did not identify any existing instruments for evaluating the use of research-based learning from an interprofessional perspective and a particular topic focus – in this case nutrition management in malnutrition – four new scales were developed. So far, the tools for evaluating IPE have mainly been for determining the learners’ attitude to IPE [13] and not the teaching method or topic used. For development of the scales, items for general teaching evaluation of the participating universities were used and new items were developed by the authors. The aim was to develop items or scales of interprofessional education linked to the topics of social climate, relevance of the topic, research-based learning and a training session evaluation in order to reflect the specific methodological and content orientation. The aim was to reduce the complexity of the training session evaluation somewhat by summarizing the items on scales.

The “IPE – Social Climate” scale (three items) was newly developed and assesses the students’ interaction with each other and the working atmosphere, also from an interprofessional perspective. The “Relevance” scale (four items) highlights the importance students attach to the interdisciplinary topic of nutrition management. The “Application of Research-based Learning” scale (five items) uses items which elicit the essential aspects of the aims and application of research-based learning, such as a systematic approach from an interprofessional perspective. The “Course Balance” scale (three items) ascertains whether the students were satisfied with the training session on the whole, if they were able to get to know the other professional group better and expand their knowledge (all items of the scales are shown in table 1 (Tab. 1)). Likert scales were used as a scaling method [14]. Answers could be given on a five point scale from “I completely disagree” (1) to “I completely agree” (5). Furthermore, socio-demographic data such as age, gender and degree program were recorded. 

It was planned to include all nursing and medical students participating in the study project in the survey.

##### 2.4.1 Checking and testing the scales

To check the internal consistency of the newly formed scales, the alpha coefficient was calculated according to Cronbach [15]. A value above 0.7 is considered acceptable [16]. The content modeling of the scales was verified by means of a principal component analysis using Varimax rotation and Kaiser normalization. The suitability of the sample for factor analysis was tested using the Kaiser-Meyer-Olkin criterion (KMO) [17]. The number of factors was determined according to the Kaiser-Guttman criterion. Only factors with an eigenvalue <1 were included. The Kaiser-Guttmann criterion is suitable because the number of items is <30, the commonalities (after extraction) average >0.7 and the sample is <200 [18]. Each item should have a factor load of at least 0.4 on the associated factor [18].

##### 2.4.2 Data analysis

The descriptive and inferential statistical analysis was carried out using the statistics program IBM SPSS-Statistics 24. In order to take into account the diversity of the students in terms of their training background to date and to obtain reliable results for the bespoke development of IPE, analyzes comparing the different professional groups were carried out after the analysis of the mean values of the four scales. For robust inferential statistical coverage of the differences between the student groups along the scales, tests for group differences for rank data according to Mann-Whitney [19] were used. The use of nonparametric tests became necessary due to the data not having a normal distribution [20] and group sizes <n=30. The a-error accumulation was counteracted through Bonferroni correction. To assess the significance of the differences, the effect size of Cohen’s d [21] was calculated for independent samples [22]. In this assessment, d=0.20 counts as a small, d=0.50 as a medium and d=0.80 as a large effect [21].

## 3. Results

### 3.1 Participants of the training session and sample of the evaluation

In the summer semester, 31 students participated in the training session (32% male); of which 14 were medical students (57% male) and 17 nursing students (12% male). In the winter semester, there were 25 participants, of which 11 were medical students (18% male) and 14 nursing students (18% male). 

In the evaluation, the summer semester 2017 and winter semester 2017/18 cohorts were merged together. This seems justifiable, as no substantial changes were made to the teaching concept after the first run. The evaluation was carried out after the last session. We were able to include 46 students, of which 21 were medical students (42.9% male) and 25 nursing students (16% male). This drop in the total number of participants in the evaluation is due to the absence of some students at the last session. One case had to be excluded due to missing answers. 

#### 3.2 Reliability of the developed scales and results of factor analysis 

A check of the scales showed good internal consistency (see table 1 (Tab. 1)). Despite the small sample size (n=46), the Kaiser-Meyer-Olkin criterion (KMO) indicates that the data are suitable for factor analysis (KMO=0.703) [23]. The exploratory factor analysis confirmed the assumed number of factors and the content modeling: All items loaded with at least 0.4 on their assigned component and with the exception of the item “All in all I am satisfied with the session” there were no double entries (see table 1 (Tab. 1)).

#### 3.3 Evaluation results

The training session run through the didactic method of research-based learning on the subject of malnutrition in case-oriented settings was rated positively by both student groups. This is reflected in particular in the excellent group score across the occupational groups, “IPE – Social Climate” (M=4.6, SD=0.76) and the estimated “Relevance” (M=4.47, SD=0.59) of the subject. The scores for “Application of Research-based Learning” (M=3.9, SD=0.72) and “Course Balance” (M=3.9, SD=0.79) show satisfactory to good results. 

**Figure 2:**
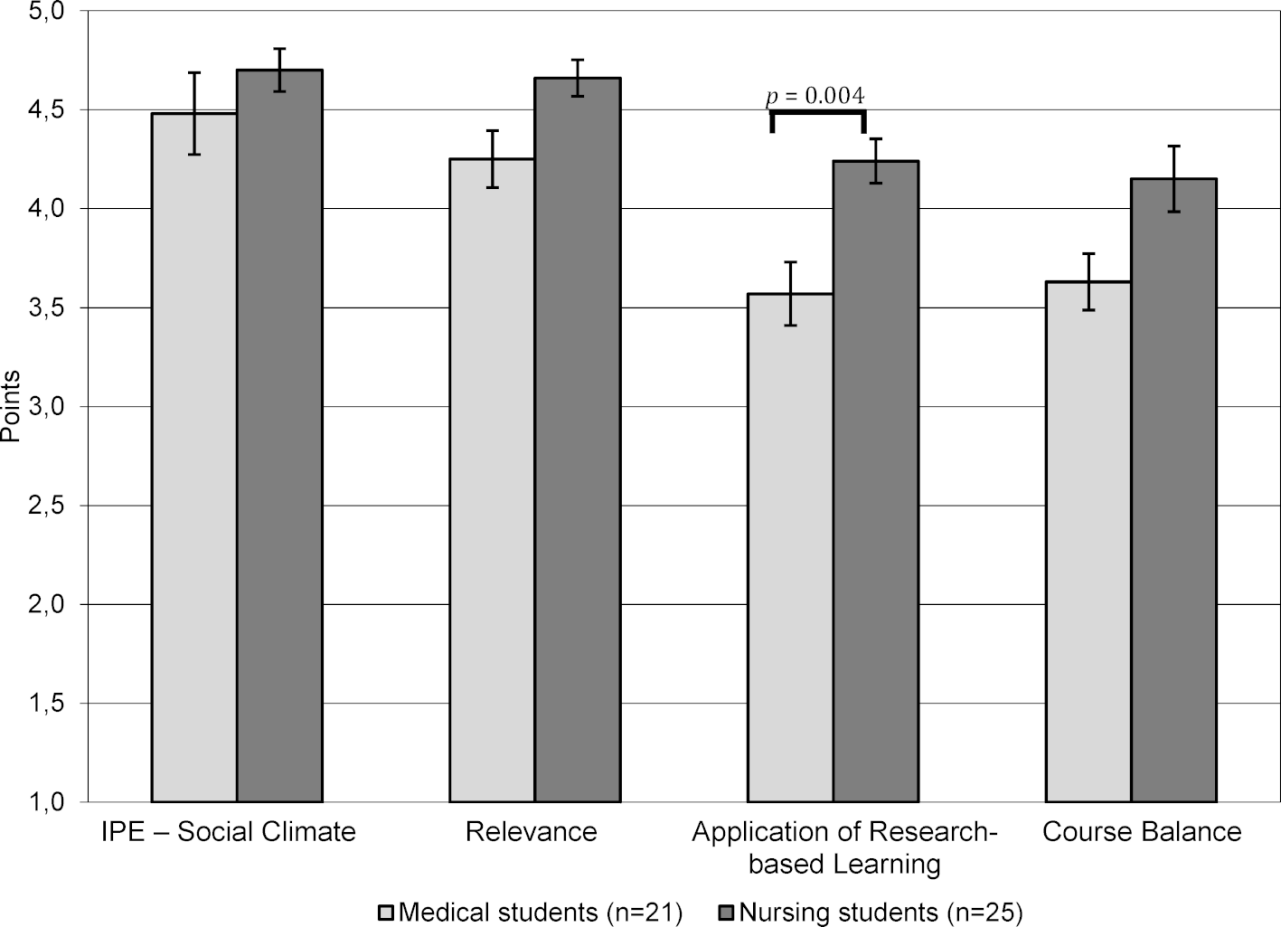
Evaluation results comparing medical with nursing students

Further analysis shows that the scales “IPE – Social Climate” (p=1.000), “Course Balance” (p=0.076) as well as “Relevance” (p=0.064) are rated positively across all occupational groups without significant differences. Only the scale “Application of Research-based Learning” (p=0.004) was rated significantly more positively by the nursing students. This mean difference shows a large effect size (d=1.04) (see figure 2 (Fig. 2)). 

## 4. Discussion

IPE, using the method of research-based learning, enabled students to try out and practice interprofessional collaboration skills in the context of nutritional management, apart from giving them a chance to practice a systematic, scientific and reflective approach. The method required close collaboration and high social contextuality in the interprofessional (research) teams, as developing an action plan and a treatment plan was only achievable through all professional groups coming together (see figure 1 (Fig. 1)). This required close consultations and working together as an interprofessional team. During the contact times, and also informally, the necessary interactions gave students the opportunity to get to know the role of the other profession. In particular the necessary close cooperation enabled an interprofessional learning environment that promoted learning about, from and with other health professionals [4]. 

In the evaluation, the training session was positively assessed by both student groups. The very positive result of the “IPE – Social Climate” scale should be stressed in particular, which constitutes an important prerequisite for interprofessional teamwork [3]. This result is particularly relevant against the background that a positive social climate and successful relationship design are prerequisites for successful training sessions. Constructivist didactics points to the primacy of relationship didactics over content didactics. “Relationships form the framework and context of any content mediation” [24]. This finding is also supported by empirical reserch into teaching and learning, which has identified a positive social climate as an important feature for high-quality teaching [25], [26].

The assessment of the topic’s relevance is also high across all professional groups. This is to be expected since there is a selection bias in the case of medical students (compulsory elective) and since it can be assumed that they are likely to have a particular interest in the subject. In nursing studies, the topic of nutritional management is dealt with in detail in the theoretical sections in any case [https://www.gesetze-im-internet.de/krpflaprv_2004/]. 

The most significant differences between the evaluation results of both student groups is on the “Application of Research-based Learning” scale, which the nursing students rated significantly higher than the medical students. In addition to the scale’s limitations that need to be discussed (see above), an explanation could be that research-based learning is a method more commonly used in nursing degrees [27], whereas in medical studies, research-based learning is pursued rather implicitly [28]. As a result, it is possible that the method was perceived as a less familiar learning culture by medical students. In addition, research-based learning requires prior knowledge of the methods and learning contents used, since the goal of the training session is to link this knowledge with high-level individual activity [8], [29]. These results point to didactic challenges as a result of different educational starting points in interprofessional training sessions [5], [30].

The “Course Balance” scale, which also shows the student’s subjective learning success, was rated satisfactorily across all professional groups. Stronger cognitive elaboration and metacognition could encourage deeper knowledge processing and increased learning among the students [31]. Metacognitive learning processes could be promoted by requiring the interprofessional teams working on the development of the joint treatment plan to justify the case-related treatment through argumentative discourse [32]. This process of targeted exchanges about different scientific approaches to the treatment of malnourished patients could also be made more prominent in the final presentation and discussion of the results. The added value of interprofessional collaboration for the treatment of patients should be highlighted as a result.

### Limitations

The relatively small number of cases in both professions should be noted as a limitation, restricting the interpretation of the data so that further evaluations of this interprofessional teaching format are necessary. In addition, it can be assumed that medical students with higher personal interest in IPE and nutrition management chose to participate (selection bias). However, this bias does not hold for the nursing students because the session was a compulsory part of the degree course. 

New scales were developed for the evaluation, as there are no instruments available for evaluation this method in the given setting. Although there are a number of validated tools for evaluating IPE [13], these primarily examine the learners’ attitudes to IPE [33] and the need for further development has been expressed due to lack of factor stability [34]. In the statistical review, the content modeling of the scales developed was shown to be reliable. The IPE-dependent items correlated highly with IPE-independent items in the scale, which indicates successful linking of the items in the respective evaluated construct. Nevertheless, the scales should be further checked for reliability and validity. This is especially true for the scale “Application of Research-based Learning”, as this is a complex multiphase teaching method. The scale did indeed map out key aspects of research-based learning but not the complete cycle. For this reason, further development and differentiation of the scale is recommended.

## 5. Conclusions

This teaching project tested the feasibility of IPE tackling the interdisciplinary topic of malnutrition through research-based learning in case-based settings and evaluated it from the students’ perspective. The teaching project was rated positively overall.

The method of research-based learning proved to be very suitable for interprofessional education, as it enabled socially contextualized learning situations which promoted the health professionals to learn from, about and with each other. 

Through the interdisciplinary case-based discussion of malnutrition, both student groups were able to develop nascent cooperation skills and capacities for action in nutrition management while still in training, providing them with realistic preparation for tasks in their future careers.

In order to promote learning success, metacognitive learning processes could be further stimulated in the students. For a systematic survey and comparison of the different education starting points, the IPE lecturers could use conditional analysis adapted to higher education didactics, similar to general didactics [35]. 

## Funding

The teaching project was funded by the Robert Bosch Foundation as part of the “Operation Team” funding line on interprofessional learning in the health professions from 2016 to 2018 (authorization number: 32.5.A381.0027.0).

## Competing interests

The authors declare that they have no competing interests. 

## Figures and Tables

**Table 1:**
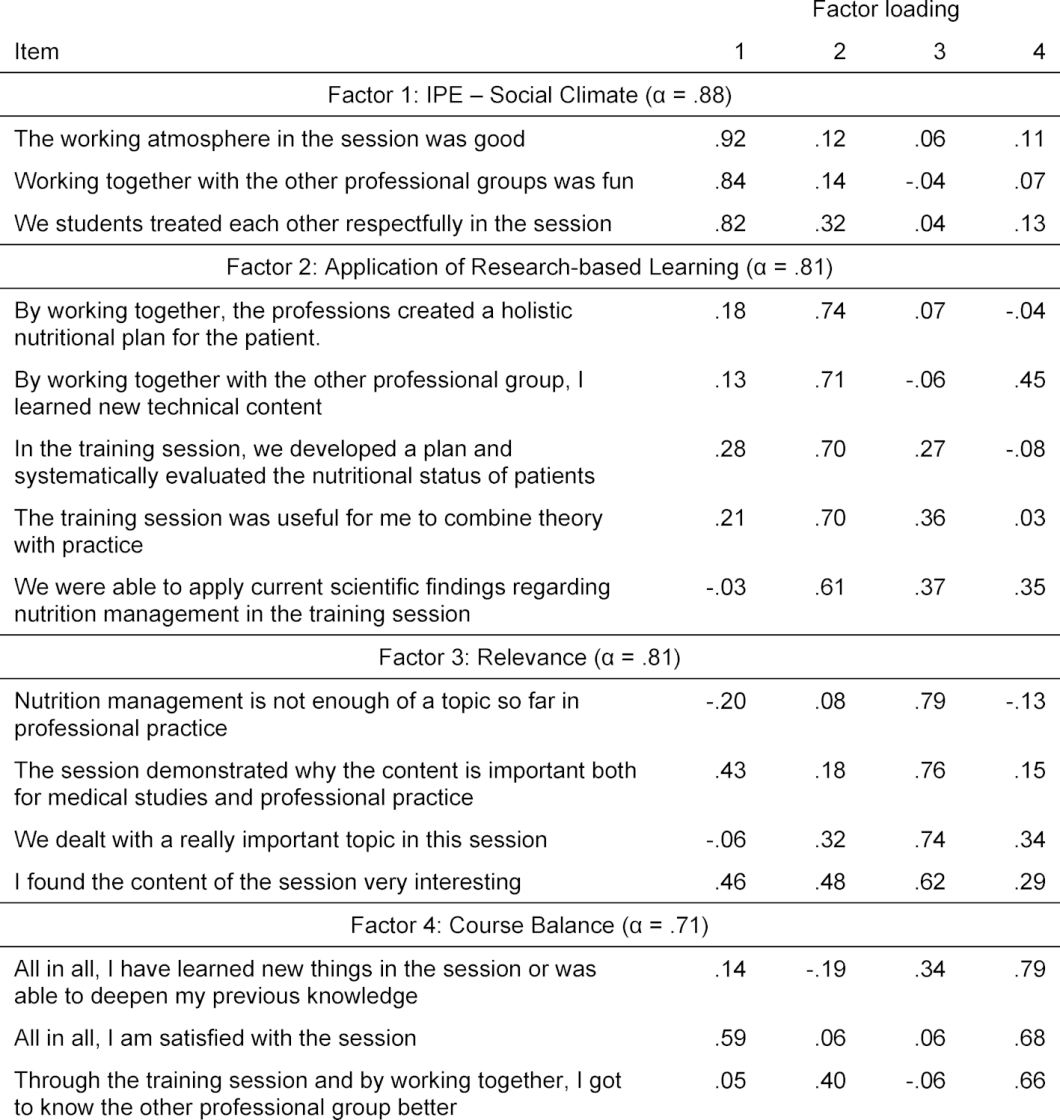
Reliability of the scales and results of factor analysis
